# Towards metrologically traceable and comparable results in GM quantification

**DOI:** 10.1007/s00216-018-1457-0

**Published:** 2018-11-06

**Authors:** Philippe Corbisier, Hendrik Emons

**Affiliations:** European Commission, Joint Research Centre (JRC), Retieseweg 111, 2440 Geel, Belgium

**Keywords:** Genetically modified organism, Unit of measurement, PCR, Traceability, Quantification, Conversion factor

## Abstract

The GM content in a food or feed product produced from or containing genetically modified organisms (GMO) has to be expressed in Europe in the form of a GM mass fraction. However, the most widely used quantification methods, based on PCR, are basically counting PCR-amplifiable DNA fragments in a sample extract. This paper outlines the requirements for obtaining comparable measurement results which are fit for regulatory decision-making. It introduces the concept of a reference measurement system which enables GMO analysis laboratories to relate their results to a universally accessible reference, thus establishing metrological traceability to a unique reference point. The conversion factors required for transforming data from one measurement unit into the other have to carry a minimum uncertainty and are anchored to specified certified reference materials. The establishment of such conversion factors and related calibration approaches to achieve comparable GM quantification results are sketched.

**Graphical abstract:**
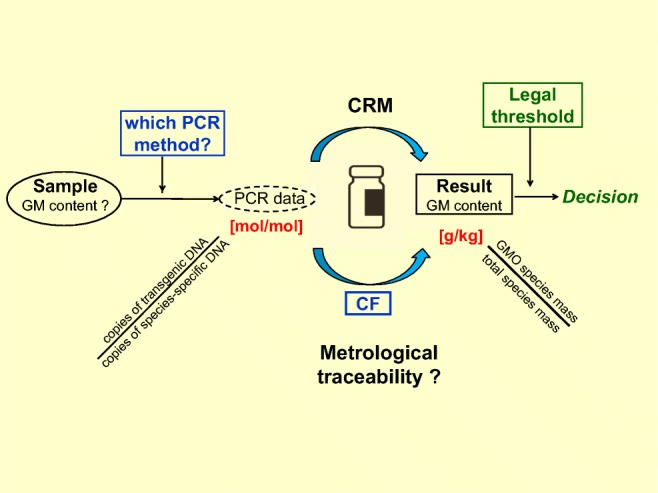
ᅟ

ᅟ

## Introduction

The prime consideration for any EU legislation on the labelling of foodstuffs is the need to inform and protect the EU consumer by providing labels that are easily understandable and accepted by consumers. Having this in mind, the measurement unit of mass already used for all ingredients in pre-packaged foodstuffs [1] was implicitly meant when introducing a labelling threshold for the genetically modified (GM) content in food and feed [2]. Later regulations made it more explicit that the GM content in a food or feed product produced from or containing genetically modified organisms (GMO) has to be expressed in Europe in the form of a GM mass fraction as stated in Regulation (EU) No. 619/2011 [3] and Regulation (EU) No. 503/2013 [4].

The most preserved analytical target through the complete food and feed chain is the DNA that can be extracted from those samples. Therefore, highly selective quantitative PCR (qPCR) methods targeting both a taxon-specific element (which is specific for the biological species) and a DNA fragment including the insertion site of the foreign DNA (GM event–specific, so-called junction sequence) have been developed for each GM event submitted to a EU market authorization process. This means that a GM measurement result which is ultimately based on the relative proportion of PCR-amplifiable taxon-specific and GM event–specific DNA fragments in a DNA extract, i.e., on the ratio of so-called DNA copy numbers, needs to be somehow transformed into a GM mass fraction.

## Expressing results

**Fig. 1:**
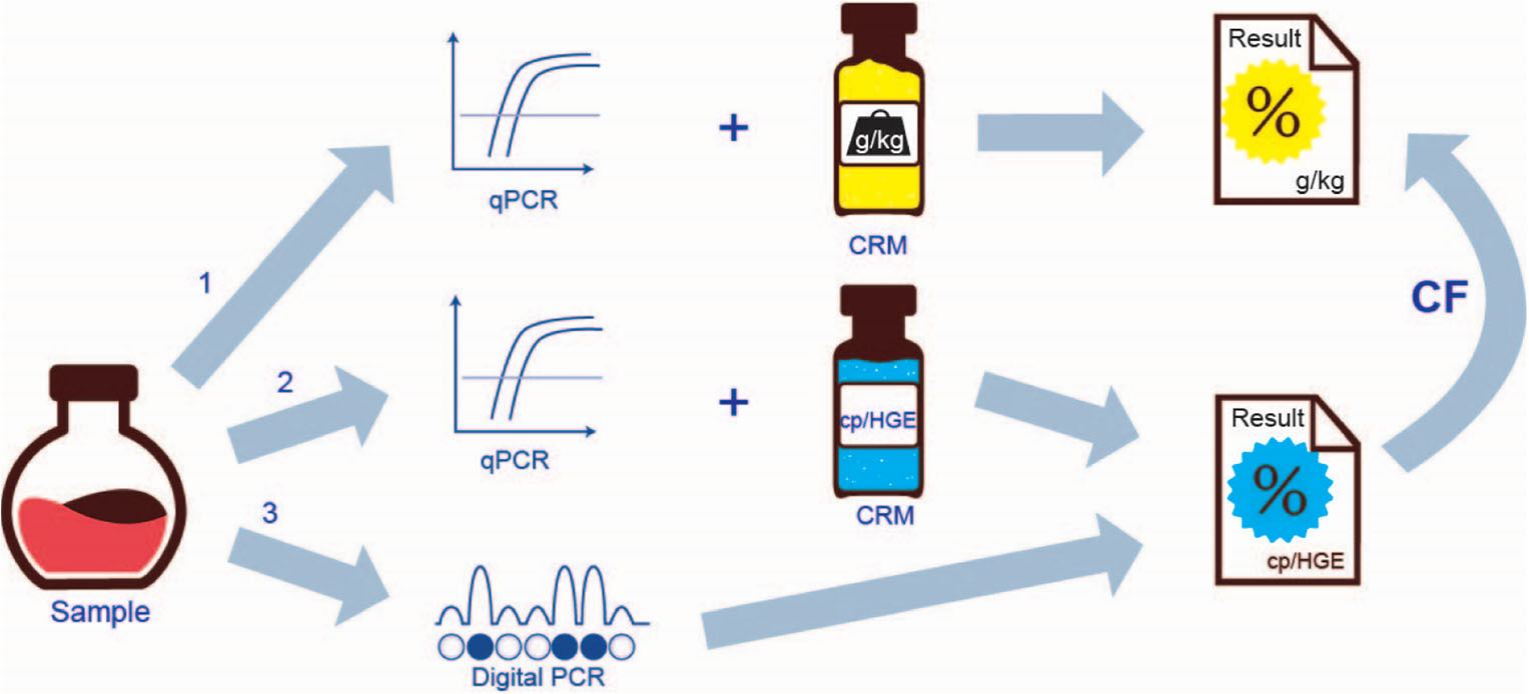
Overview of the various options to measure the GM content present in a sample by PCR. CF, conversion factor (adapted from [5])

An exact mathematical relationship between the DNA copy number ratio and the corresponding mass fraction does not exist. Consequently, there are basically three ways to proceed (Fig. 1).

The first option consists in measuring the DNA extracted from the product by two qPCR methods, one being taxon-specific and one being GM event–specific. Both methods need to be calibrated with DNA extracted from a certified reference material (CRM) containing a known mass fraction of the GM. Such a calibration approach implies an ‘invisible’ conversion between DNA fragments, contained in the weighed-out material components during CRM preparation, and the mass fraction stated by the CRM producer on the certificate. By following this route, the result obtained is automatically expressed in the measurement unit of the certified property value of the CRM, i.e., in g/kg.

The second option (Fig. 1) involves measuring the DNA extracted from the sample by the same qPCR methods as mentioned above, but to calibrate them with DNA CRMs which have been certified for their GM copy number ratios. The CRM could either be a linearized plasmid DNA that contains both taxon-specific targets and junction-specific targets in a known ratio or a matrix material, such as ground seeds, which has been certified to contain a particular GM content expressed as the ratio of GM sequences per DNA copies of a particular species. In this case, the results will be expressed as the percentage of GM DNA copy numbers in relation to the taxon-specific DNA copy numbers calculated in terms of haploid genomes. Consequently, the result still needs to be converted into a GM mass fraction.

The third option (Fig. 1) results from measuring the DNA extracted from a product by digital PCR (dPCR). In this case, the PCR methods do not require particular DNA calibrators. The result will be based on the ratio of the absolute number of GM event–specific and taxon-specific amplifiable copies that have been counted. As for option 2, this ratio needs to be converted into a GM mass fraction.

## Transforming quantities

Obviously, it would be most convenient, if a single conversion factor (CF) could be used for transformations between the two measurement scales. Proposals have been made (see, for instance, [6]) to apply ‘universal’ CFs for each biological species taking into account its zygosity. However, the unknown influence of biological factors contributing to the genetic composition of the food ingredient, such as the parental origin of the transgene and the degree of mixture of different tissue types of the same species in the sample, introduces a large uncertainty into the application of universal species-related CFs. Therefore, another approach for establishing conversion factors which enable achieving metrological traceable and comparable measurement results in GM quantification has been introduced [5]. It is based on the concept of reference measurement systems, composed of a combination of reference materials and reference measurement procedures. This concept had been already introduced for the measurement of clinical biomarkers and allows now the global comparability of measurement results for complex measurands in clinical chemistry and laboratory medicine [7, 8]. In an analogous manner, the reference system for GM quantification can make use of specified CRMs. The GM event–specific conversion factor as the ratio of the number of DNA copies of the transgenic sequence divided by the number of DNA copies of the species-specific sequence is then determined with that CRM to anchor the reference system to one of the base units of the International System of Units (SI), namely the kg.

## Establishing conversion factors

The concept of converting results for GM quantification from one unit, i.e., measurement scale, into another one can be realized when the following three requirements are put into practice for each GM event:

- a unique reference point for defining the CF is available;
- the CF value carries a minimum uncertainty;
- the uncertainty of the CF value is included into the uncertainty of the final result.

The first aspect is fundamental for establishing metrological traceability of the measurement results to such an extent that data originally created at the copy number ratio or mass fraction scales are made comparable. Such a traceability can currently only be ensured by using a single CRM as a unique ‘artificial’ anchoring reference point. With a CRM as calibrant, which has been gravimetrically prepared from pure GM and pure non-GM starting materials and is certified for its mass fraction regarding a specified GM event, the qPCR measurement result is anchored to this quantity value embedded in that particular CRM. The traceability chain for the property value of a CRM certified for its GM mass fraction is based on the use of calibrated balances and a thorough control of the weighing and mixing procedures [9]. The certified value is therefore traceable to the kg as one of the base units of the SI. If another CRM would be used for qPCR calibration (having the same GM mass fraction but, e.g., a different number of transgenic or species-specific genes), a different result would be obtained. Therefore, the CRM providing the reference for the GM event–specific CF has to be unique and widely available.

As a matter of fact, different CRM types and matrices have been developed over the years, also because the measurement unit was not unanimously agreed at national and international levels. For instance, CRMs have been produced of pure milled or intact GM seeds and were certified for the presence of a GM event, specifying ‘purity’ for the homozygous or heterozygous GM event [10]. Usually, such materials have to be considered as containing 1000 g GM material per kg; however, this is often not stated on the certificate. There are also CRMs consisting of a mixture of milled GM seeds and milled non-GM seeds which have been certified to contain a certain mass fraction of GM material in the total mass [11]. A limited number of these materials were additionally certified for the copy number ratio between incorporated GM DNA fragments and taxon-specific DNA fragments [12]. In some cases, leaves, which contain a more uniform tissue with respect to zygosity in comparison to seeds, have been used to prepare DNA CRMs certified for the presence of a GM event [13]. Moreover, a few dual-target plasmids containing a single copy of both the GM event–specific and the taxon-specific target have been certified [14]. These plasmid DNA (pDNA) solutions can be used to calibrate qPCR experiments. However, they provide a different reference point for metrological traceability, i.e., they establish a different reference system compared to the system based on extracted genomic DNA (gDNA). Indeed, notwithstanding that the commutability of pDNA has been demonstrated for some GM measurement procedures [15], small differences in PCR efficiency have been observed for gDNA and pDNA measured with other procedures [16], which means that the result is only traceable to the DNA ratio of the particular DNA calibrant used.

Despite that various calibrators for the same GM event may be available, the choice of the CRM to be used for creating a more universal reference system including the establishment of a CF is quite straightforward. Indeed, the EU register of authorized GMOs, which is listing the products registered, withdrawn or pending EC decisions for each GM event, does not only provide the official method of GM detection but also provides the name and code of the CRM that has been made available for official market controls. This information is part of the legal basis authorizing the placing of products containing, consisting of, or produced from, a particular GM event on the EU market. The CRM specified in legislation is available for analytical laboratories and can serve as universal anchor for defining the CF per GM event. In practice, most official control laboratories are using or extracting the gDNA for the purpose of generating calibration curves from the CRM containing the highest mass fraction of a particular GM event in the battery of CRMs with different mass fractions for this event. Therefore, the conversion factor shall be determined on that particular CRM and not on any other material that may contain the same GM event. CRM producers avoid offering two concomitant batches, produced from different starting materials, of a CRM for the same GM event. When a new batch of a particular CRM is released, the older batch is removed from sale and the expiry date of the corresponding certificate is not renewed. Thereby, only one valid CRM per GM event can be used by a laboratory.

The second requirement listed above is favouring analytical strategies with which the determination of the CF could be directly performed by measuring the CRM without introducing another calibration step. A determination of the CF by qPCR using, for example, a dual-target pDNA as calibrant would not only introduce another traceability chain and another reference system but also increase the uncertainty accompanying the CF by additional uncertainty contributions. A more straightforward approach consists in determining the CF directly by dPCR, as this DNA quantification technique does not require a particular calibrant. The dPCR procedures applied to fix the CF should target the DNA sequences that have been demonstrated to be specific for a particular GM event and a particular biological species. In other words, the forward/reverse primers and probes referred to in the qPCR method validation reports issued by the EU Reference Laboratory for Genetically Modified Food and Feed (EURL-GMFF) in the frame of the market authorizations [17] should also be used in the dPCR procedure because the identity of the measurand has to be maintained.

For expressing the result of a GM quantification after converting PCR measurement data with the help of the CF to the mass fraction scale, the stated uncertainty has to include, besides the uncertainty components of the actual analytical procedure, also the uncertainty of the CF. Therefore, this uncertainty contribution has to be known and should not significantly enlarge the combined uncertainty for still allowing a meaningful decision about product compliance with legal thresholds.

## Comparable results

By respecting those three requirements for fixing and applying conversation factors, a reference measurement system for comparable GM quantification results can be established. It is for each GM event composed of the validated qPCR method, which is published by the EURL-GMFF, and the respective CRM listed in legislation. The latter can either be directly used as calibrator for qPCR measurements, in case that materials with appropriate GM mass fractions are available or could be prepared in-house with sufficient accuracy (option 1 in Fig. 1), or the CRM would serve as reference for the determination of the GM event–specific CF, which is then used to convert measurement data obtained in the form of DNA copy numbers into the final result expressed as GM mass fraction (options 2 and 3 in Fig. 1).

By following this approach, measurement results expressing the GM content in a food or feed product are traceable to a unique reference system and would be comparable, independent of the PCR technique applied, and in line with EU legislation.

An interlaboratory exercise has been launched at the European level to determine by dPCR the CF of 52 single GM events currently authorized in the EU. As a result, the already available CRMs for each GM event should be accompanied by unique conversion factors. The concept of linking a measurement result to a particular reference material and a well-defined reference method for enabling comparability could be further expanded to the quantification of other complex analytes such as allergens, where a complete reference measurement system still needs to be established.
